# Real-world effectiveness of sotrovimab for the treatment of SARS-CoV-2 infection during Omicron BA.2 and BA.5 subvariant predominance: a systematic literature review

**DOI:** 10.1007/s15010-024-02245-6

**Published:** 2024-04-11

**Authors:** Myriam Drysdale, Mehmet Berktas, Daniel C. Gibbons, Catherine Rolland, Louis Lavoie, Emily J. Lloyd

**Affiliations:** 1 Value Evidence and Outcomes, GSK, 980 Great West Road, Brentford, Middlesex TW8 9GS UK; 2 Evidence Synthesis, Modelling and Communications, PPD Evidera, London, UK; 3 Evidence Synthesis, Modelling and Communications, PPD Evidera, Montreal, Canada

**Keywords:** COVID-19, Omicron BA.2, Omicron BA.5, Sotrovimab, Hospitalizations, Mortality

## Abstract

**Purpose:**

To evaluate clinical outcomes associated with sotrovimab use during Omicron BA.2 and BA.5 predominance.

**Methods:**

Electronic databases were searched for observational studies published in peer-reviewed journals, preprint articles and conference abstracts from January 1, 2022 to February 27, 2023.

**Results:**

The 14 studies identified were heterogeneous in terms of study design, population, endpoints and definitions. They included > 1.7 million high-risk patients with COVID-19, of whom approximately 41,000 received sotrovimab (range *n* = 20–5979 during BA.2 and *n* = 76–1383 during BA.5 predominance). Four studies compared the effectiveness of sotrovimab with untreated or no monoclonal antibody treatment controls, two compared sotrovimab with other treatments, and three single-arm studies compared outcomes during BA.2 and/or BA.5 versus BA.1. Five studies descriptively reported rates of clinical outcomes in patients treated with sotrovimab. Rates of COVID-19-related hospitalization or mortality (0.95–4.0% during BA.2; 0.5–2.0% during BA.5) and all-cause mortality (1.7–2.0% during BA.2; 3.4% during combined BA.2 and BA.5 periods) among sotrovimab-treated patients were consistently low. During BA.2, a lower risk of all-cause hospitalization or mortality was reported across studies with sotrovimab versus untreated cohorts. Compared with other treatments, sotrovimab was associated with a lower (molnupiravir) or similar (nirmatrelvir/ritonavir) risk of COVID-19-related hospitalization or mortality during BA.2 and BA.5. There was no significant difference in outcomes between the BA.1, BA.2 and BA.5 periods.

**Conclusions:**

This systematic literature review suggests continued effectiveness of sotrovimab in preventing severe clinical outcomes during BA.2 and BA.5 predominance, both against active/untreated comparators and compared with BA.1 predominance.

**Supplementary Information:**

The online version contains supplementary material available at 10.1007/s15010-024-02245-6.

## Introduction

As of October 2023, there have been over 770 million confirmed cases of COVID-19 globally, including nearly 7 million deaths [1]. Since the declaration of the COVID-19 pandemic by the World Health Organization (WHO) in March 2020 [2], new severe acute respiratory syndrome coronavirus 2 (SARS-CoV-2) variants have continued to emerge [3, 4]. COVID-19 continues to be responsible for a substantial number of new infections globally, placing a strain on healthcare systems around the world [1, 5].

Sotrovimab is a dual-action recombinant human IgG1κ monoclonal antibody (mAb) derived from the parental mAb S309, a potent neutralizing mAb directed against the spike protein of SARS-CoV-2 [6–9]. The safety and efficacy of sotrovimab was demonstrated in the pivotal COMET-ICE randomized clinical trial (NCT04545060), conducted during the original ‘wild-type’ variant period of the pandemic [10]. A single intravenous (IV) infusion of sotrovimab (500 mg) was found to significantly reduce the risk of all-cause > 24-h hospitalization or death by 79% compared with placebo in a high-risk population with COVID-19 [10]. Sotrovimab (IV 500 mg) was subsequently granted Emergency Use Authorization (EUA) by the United States (US) Food and Drug Administration (FDA) for the treatment of mild-to-moderate COVID-19 in adults and pediatric patients (≥ 12 years of age and ≥ 40 kg) who tested positive for SARS-CoV-2 and were at a high risk of progression to severe COVID-19, including hospitalization or death [11]. Sotrovimab was also granted marketing authorization in the European Union, Norway and Iceland [12], and Bahrain, and conditional marketing authorization in Australia [13], the United Kingdom [14], Saudi Arabia and Switzerland [15]. In Japan, a Special Approval in Emergency has been granted, and temporary/emergency authorizations were granted in Canada, and the United Arab Emirates.

Since the COMET-ICE trial was undertaken, new viral variants have emerged, including the Omicron BA.2 subvariant that became predominant globally in March 2022 and the BA.5 subvariant that became predominant in August 2022 [16, 17]. In vitro neutralization assays demonstrated that sotrovimab retained its neutralization capacity against Omicron BA.1 but showed reduced neutralization potency against later variants, such as Omicron BA.2 and BA.5 (16- and 22.6-fold changes in EC_50_, respectively) [18]. In the absence of clinical trials to assess the efficacy of sotrovimab against these emerging variants, the clinical relevance of this reduced neutralization observed in vitro was unknown, and the FDA took the decision in April 2022 to deauthorize the EUA for sotrovimab in the US [19].

Generating near real-time data on the efficacy of sotrovimab in the constantly evolving SARS-CoV-2 variant landscape is challenging, and there is no validated model that can reliably correlate in vitro neutralization to predicted clinical efficacy; hence, real-world evidence is a key source of information to assess the benefit-risk profile of sotrovimab. A published systematic literature review (SLR) and meta-analysis of 17 studies including 27,429 patients concluded that sotrovimab is an effective and well-tolerated therapy that can reduce mortality and hospitalization rates in patients infected with both the Delta and Omicron BA.1 variants [20]. In addition, we previously conducted a SLR of papers published from January 1st to November 3rd, 2022, the results of which suggested continued clinical effectiveness of sotrovimab in preventing severe clinical outcomes related to COVID-19 during Omicron BA.2 predominance versus a control/comparator and compared with the period of BA.1 predominance [21].

To investigate the use of sotrovimab against emerging variants among patients either partially or fully vaccinated against or previously exposed to SARS-CoV-2, including impact on clinical outcomes, a SLR was undertaken to evaluate the current evidence on the clinical effectiveness of sotrovimab during Omicron BA.2 and BA.5 predominance. This SLR builds on our previous review [21] to cover studies including BA.5 predominance periods and newly published papers on BA.2.

## Methods

This SLR included observational studies investigating clinical outcomes in patients treated with sotrovimab published in peer-reviewed journal articles, preprint articles, and conference abstracts between January 1, 2022 and February 27, 2023. The publication period was selected to identify publications reporting data during Omicron BA.2 and BA.5 predominance. Where available, data on other circulating variants were also extracted for potential comparison between periods of variant predominance.

The SLR was conducted in accordance with Preferred Reporting Items for Systematic Reviews and Meta-Analyses (PRISMA) guidelines (PROSPERO registration number: CRD42022376733) [22].

### SLR objectives

The primary objective of the SLR was to assess the clinical effectiveness of sotrovimab in patients receiving early treatment for COVID-19 (as used in accordance with local COVID-19 guidelines) during the Omicron BA.2 and BA.5 predominance periods.

### Data sources and search strategy

Searches were conducted using the following indexed electronic databases: MEDLINE (via OVID), Embase (via OVID), LitCovid (via MEDLINE), Cochrane COVID-19 Study Register, and EconLit. Additional searches for relevant preprints were conducted in ArRvix, BioRxiv (via Embase), ChemRvix, MedRxiv (via Embase), Preprints.org, ResearchSquare, and SSRN.

The following conferences were also searched for relevant abstracts indexed from January 1, 2022: Infectious Diseases Week; International Conference on Emerging Infectious Diseases; European Respiratory Society; and European Congress of Clinical Microbiology and Infectious Diseases. These conferences were selected as they were likely to include a wide range of newly available research in the field of COVID-19 therapeutics and management.

Search strategies, starting from January 1, 2022 for each database, included a combination of free-text search terms for COVID-19, different variants, sotrovimab, and observational study design (Supplementary Table 1). There was no limit on geographical location, but only English language publications were considered.

### Study selection

Studies were screened and selected for inclusion in the SLR against predetermined PICOS (populations, interventions and comparators, outcomes, and study design) criteria [23]. Only studies matching any inclusion criteria and none of the exclusion criteria listed in Table 1 were eligible for inclusion in the review. As the focus of this SLR was on outcomes captured during Omicron BA.2 and BA.5 predominance periods, only papers reporting these subvariants are included here.

**Table 1:** Inclusion and exclusion criteria

Domain	Criteria	Exclusion reason	Exclusion description
Populations	Patients aged ≥ 12 years who fulfill the following criteria: Identified as having confirmed COVID-19 Have received sotrovimab for treatment of SARS-CoV-2 infection as per standard of care Presented with the BA.2 subvariant onwards, or had COVID-19 during BA.2 subvariant and onwards dominant period Subgroups of interest: Subgroup within high-risk group (i.e., transplant patients, renal patients)	Population not of interest	Patients aged < 12 years
Interventions/comparators	All studies with patients treated with sotrovimab 500 mg IV (*n* ≥ 20)	No treatment of interest	Did not receive sotrovimab Received sotrovimab as a prophylactic treatment, or for primary treatment of severe COVID-19 Fewer than 20 patients treated with sotrovimab
Outcomes	Any of the following clinical outcomes within 30 days of sotrovimab: Hospitalization and/or mortality (all-cause or COVID-19-related) Intensive care admission Emergency department visits Respiratory support (e.g., use of supplemental oxygen) COVID-19 progression (e.g., composite endpoint such as ICU/respiratory support/mortality)	Outcomes not of interest	Relevant outcomes are not reported
Study design	Any of the following study designs: Observational studies (including sotrovimab-treated single-arm studies and comparative effectiveness studies) SLRs with or without meta-analysis (for citation chasing of observational studies only)	Publication type not of interest Study design not of interest	Case report, editorial, opinion piece, letter to the editor, clinical trial, narrative review, guidelines Pre-clinical studies (animal, in vitro, ex vivo, pharmacokinetics) Clinical trials

*COVID-19* coronavirus disease 2019, *ICU* intensive care unit, *SARS-CoV-2* severe acute respiratory syndrome coronavirus 2, *SLR* systematic literature review

Two independent reviewers evaluated each title and abstract against the defined selection criteria to determine suitability for the SLR, with disagreements resolved by a third reviewer. The same process was applied for the review of the full-text articles.

### Data extraction and quality assessment

Extraction of data from the included studies was performed by a single extractor using a data extraction file designed in Microsoft Excel. An independent researcher reviewed all extracted fields, with discrepancies resolved by a third reviewer.

Extracted information included the study title and reference, study details and design, country(ies), data source, study population, number of patients, data collection period and associated circulating SARS-CoV-2 variants, follow-up duration, sponsor, key baseline characteristics, and clinical outcomes. Clinical outcomes included hospitalization and/or mortality, intensive care admission, emergency department visits, respiratory support (e.g., use of supplemental oxygen), and COVID-19 progression (e.g., composite endpoint such as intensive care unit [ICU]/respiratory support/mortality).

The 8-item Newcastle Ottawa Scale (NOS) was used to assess the quality of each study by considering characteristics that could introduce bias [24, 25]. Studies were assessed based on three broad domains of their design: (1) selection of study groups, (2) comparability of the participants in each group, and (3) ascertainment of either the exposure or outcome of interest for case–control or cohort studies, respectively [24]. For each study, the maximum attainable score in a NOS quality assessment is nine (accumulated across all domains), with greater scores representing a lower risk of bias.

## Results

### Study selection

**Fig. 1:**
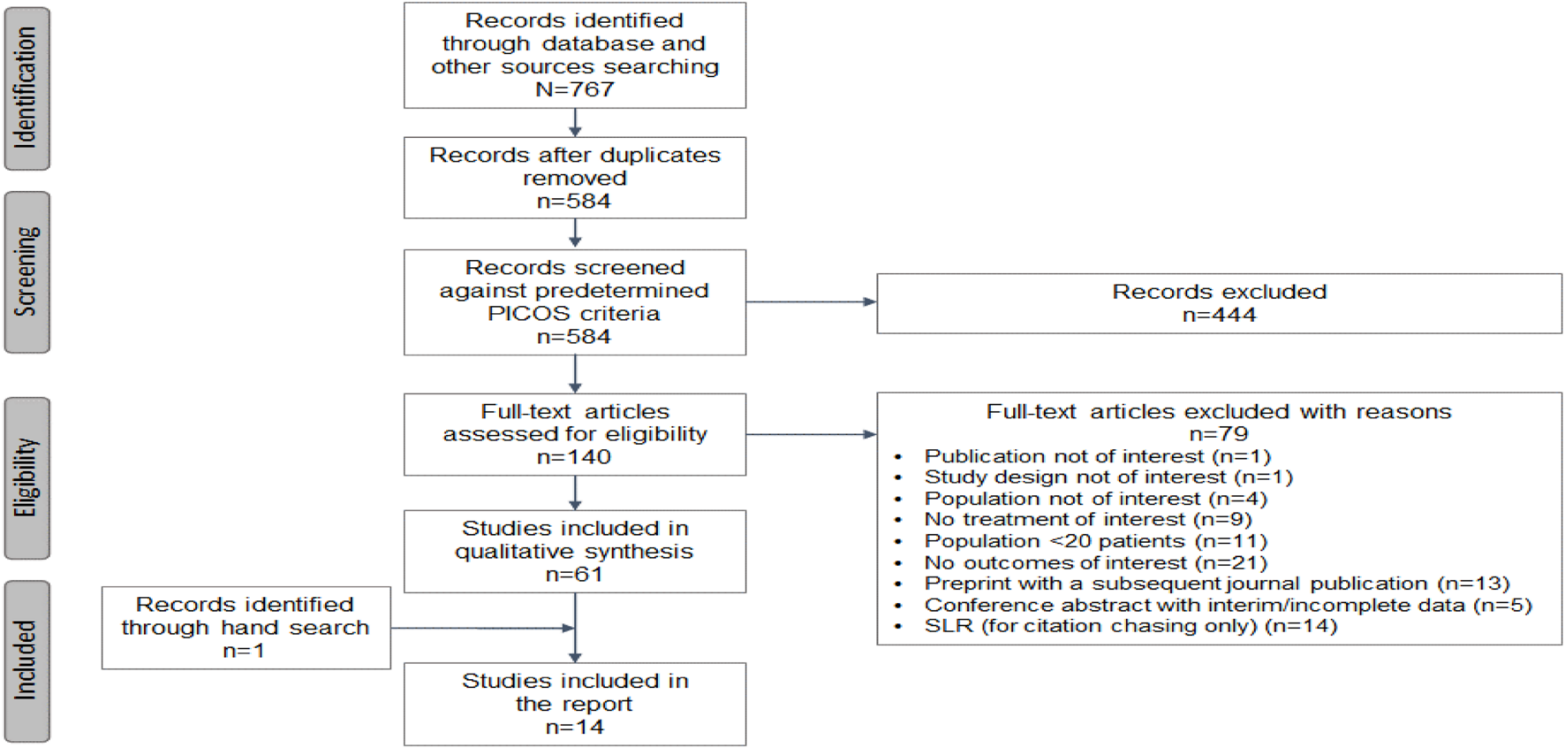
PRISMA flow diagram of studies included in the SLR. *PICOS* populations, interventions and comparators, outcomes, and study design *PRISMA* Preferred Reporting Items for Systematic Reviews and Meta-Analyses, *SLR* systematic literature review

Searches from electronic database and conference abstracts, preprints and citation chasing from relevant SLRs yielded a total of 767 papers (Fig. 1). After removal of duplicates, 584 unique titles and abstracts were screened, of which 140 were considered admissible for full-text review. Of these, 14 contained clinical outcome data for sotrovimab from the BA.2 and BA.5 periods onwards and were determined eligible for inclusion in the SLR. Reasons for exclusion during the full-text review are detailed in Fig. 1.

### Study characteristics

An overview of the key characteristics of the 14 observational studies included in the SLR is provided in Table 2.

**Table 2:** Overview of characteristics of studies included in the SLR

Author, year	Country (region)	Study design/clinical outcomes assessed	Analytical methods summary	Data source	Study time period	Stated BA.2 and BA.5 prevalence (%) during time period (ecological studies)	Population	Sotrovimab/comparator	Sample size (*N*)	BA.2 and BA.5 sample size (*N*)	Key baseline characteristics
Cheng et al., 2023 [26] (peer- reviewed)	US (all)	Observational comparative effectiveness cohort study All-cause hospitalization within 30 days of claimed COVID-19 diagnosis; 30-day faculty-reported all-cause mortality; composite of 30-day all-cause hospitalization or mortality	Multivariate and propensity score matched (1:4) regression analyses	FAIR Health claims database	Sept 1, 2021 to Apr 30, 2022	Monthly average US prevalence: Mar 22: ~ 50% Apr 22: ~ 100%	High-risk patients (based on EUA criteria) diagnosed with COVID-19	Sotrovimab (S) No mAb	S: 15,633 No mAb: 1,514,868 (62,532 for matched cohort)	**BA.2** S: 1,114 No mAb: 182,759 (Ecological) Mar 1 to Apr 30, 2022	Immunocompromising conditions/immune-suppressive therapy S: 6,525 (41.7%) No mAb: 379,002 (25.0%) Documented COVID-19 vaccine S: 3177 (20.3%) No mAb: 229,770 (15.2%)
Evans et al., 2023 [33] (preprint at time of search; now peer-reviewed)	UK (Wales)	Observational comparative effectiveness cohort study All-cause hospitalization or death	Cox regression analyses	Secure Anonymised Information Linkage (SAIL) databank	Dec 16, 2021 to Apr 22, 2022	NR	High-risk non-hospitalised adult patients with COVID-19 using the SAIL databank	Sotrovimab (S) Molnupiravir (M) Nirmatrelvir/ritonavir (Nir/Rit) Untreated (U)	Total: 7,103 S: 1,079 M: 359 Nir/Rit: 602 U: 4,973	NR	Immunosuppressed: Treated: 968 (47.5%) Untreated: 2,042 (41.1%) ≥ 4 vaccine doses: Treated: 740 (36.3%) Untreated: 875 (17.6%)
Fujimoto et al., 2022 [27] (peer-reviewed)	Japan (Kishi-wada)	Observational comparative effectiveness cohort study Mortality and requirement for ICU or oxygen therapy	Descriptive analysis for clinical outcomes	Kishiwada City Hospital	July 24, 2021 to Aug 10, 2022	BA.5: 100% during BA.5 period (July 1 to Aug 10, 2022)	COVID-19 patients hospitalized during delta and omicron subvariants BA.1 and BA.5 periods, treated with sotrovimab, casirivimab/imdevimab or remdesivir and dexamethasone with or without baricitinib	Sotrovimab (S) Casirivimab/imdevimab (Cas/Imd) Remdesivir (R) Dexamethasone ± baricitinib (double or triple therapy)	179	**BA.5** 76 (total) S: 47 Triple Rx: 17 Double Rx: 12 (Ecological) July 1 to Aug 10, 2022	40 vaccinated and 2 unvaccinated patients received sotrovimab
Harman et al., 2022 [34] (preprint at time of search; now peer-reviewed)	UK (England)	Observational comparative cohort study All-cause hospital admission	Stratified Cox regression	UKHSA	Jan 1, 2022 to Apr 26, 2022	Variant confirmed by laboratory data	High-risk patients with confirmed SARS‐CoV‐2 Omicron BA.1 and BA.2 treated with sotrovimab in the community	Sotrovimab BA.2 confirmed infected patients vs Sotrovimab BA.1 confirmed infected patients	BA.2: 4565 BA.1: 4285	**BA.2** 4,565	≥ 14 days after second COVID-19 vaccine dose BA.1: 4136 (96.5%) BA.2: 4432 (97.1%)
Martin-Blondel, et al., 2023 [28] (peer-reviewed)	France (all)	Observational comparative effectiveness cohort study COVID-19-related hospitalization or death	Descriptive analysis for clinical outcomes Multivariable Cox regression analysis	Ongoing ANRS 0003S CoCoPrev study	Jan 24, 2022 to May 5, 2022	Confirmed variants with sequencing data	Patients at high-risk for progression with mild-to-moderate BA.1 or BA.2 COVID-19	Sotrovimab (S) Nirmatrelvir (N)	255	**BA.2** Total: 92 (Sequence-confirmed)	Immunosuppressive therapy: S: 55 (38%) N: 9 (26%) ≥ 3 doses vaccine: S: 147 (78%) N: 52 (87%)
Mazzotta et al., 2023 [29] (peer-reviewed)	Italy (Rome)	Observational comparative cohort study Hospitalization due to severe COVID-19 or death from any cause	Descriptive analysis for clinical outcomes	Single center (primary data collection)	Jan 1, 2022 to Apr 26, 2022	Confirmed variants with sequencing data	Outpatients with sequence confirmed SARS‐CoV‐2 Omicron (BA.1 or BA.2) diagnosis and a mild‐to‐moderate COVID‐19 (AIFA eligibility criteria)	Sotrovimab (S) Molnupiravir (M) Remdesivir® Nirmatrelvir/ritonavir (Nir/Rit)	S: 202 M: 117 R: 118 Nir/Rit: 84	**BA.2** S: 56 M: 18 R: 34 Nir/Rit: 35 (Sequence-confirmed)	Primary/secondary immunodeficiency S: 52 (25.7%) M: 17 (14.5%) R: 18 (15.3%) Nir/Rit: 10 (11.9%) Partly or fully vaccinated S: 182 (91.0%) M: 108 (93.1%) R: 101 (85.6%) Nir/Rit: 78 (92.9%)
Nose et al., 2022 [32] (peer-reviewed)	Japan (All)	Observational comparative effectiveness cohort study Progressor rate^c^	Descriptive analysis for clinical outcomes	Ongoing multicentre observational study (interim analysis)	Jan 31, 2022 to Aug 19, 2022	BA.2: 5.8% (*n* = 20/346)^d^ during March 28 to June 19, 2022	Patients infected with SARS‒CoV-2, with risk factors for progression to severe infection, not requiring oxygen therapy at baseline, receiving sotrovimab for the first time	Sotrovimab	346 (246 in clinical outcomes analysis)	**BA.2** 20^d^	Immunosuppressive disease or treatment: Total 22 (6.4%) Number of vaccine doses (*n* = 162 patients): 1 dose: 9 patients 2 doses 85 patients 3 doses: 68 patients
Patel et al., 2022 [36] (preprint)	UK (England)	Observational comparative effectiveness cohort study COVID-19-related and all-cause hospitalization; all-cause death	Descriptive analysis for clinical outcomes	Discover-NOW dataset	Dec 1, 2021 to May 31, 2022^b^	BA.2: 90.1% sequenced cases across England during BA.2 period (Mar 1, 2022 to May 31, 2022) BA.5: 70.6% during BA.5 period (June 1, 2022 to July 31, 2022)	COVID-19 patients treated with sotrovimab, nirmatrelvir/ritonavir or molnupiravir, or patients at highest risk per NHS criteria but who were untreated	Sotrovimab (S) Nirmatrelvir/ritonavir (Nir/Rit) Molnupiravir (M) Remdesivir (R) Untreated (U)	Total period: 5547 S: 696 Nir/Rit:337 M: 470 U: 4044	**BA.2 (total)** 2045 S: 415 Nir/Rit:269 M: 59 U:1302 **BA.5 (total)** 1,095 S: 197 Nir/Rit:228 M: 13 U: 657 (Ecological) March 1 to May 31, 2022 for BA.2; June 1 to July 31, 2022 for BA.5	Immune deficiencies S: 50 (7.2%) Nir/Rit: 96 (28.5) M: 47 (10.0) U: 1080 (26.7) > 1 booster vaccine S: 238 (34.2%) Nir/Rit: 102 (30.3%) M: 78 (16.6%) U: 553 (13.7%)
Patel et al., 2023 [35] (preprint)	UK (England)	Observational comparative effectiveness cohort study COVID-19-related hospitalization; all-cause hospitalization or death	Multivariate Poisson regression analyses	Hospital Episode Statistics database	Jan 1, 2022 to July 31, 2022	BA.2 ≥ 75% during period 3 (Feb 28 to May 1, 2022) BA.5 ≥ 75% during period 6 (July 4 to July 31, 2022)	High-risk patients with COVID-19 presumed treated with sotrovimab in NHS hospitals across England	Sotrovimab	10,096	**BA.2** ≥ 75% prevalence (Period 3): 3884 **BA.5** ≥ 75% prevalence (Period 6): 1383 (Ecological)	Immunosuppressed: Total: 338 (3.3%)
Rasmussen et al., 2023 [30] (peer-reviewed)	Denmark (all)	Observational comparative effectiveness cohort study Hospitalization or all-cause death	Cox regression Analyses Additional sensitivity analyses	Danish Civil Registration System, Danish National Hospital Registry, Danish Vaccination Registry, National COVID-19 Surveillance System, Danish COVID-19 Genome Consortium	Sept 6, 2021 to July 1, 2022	1,573/2,933 (53.6%)	High-risk group individuals treated with sotrovimab following a positive SARS-CoV-2v test in Denmark	Sotrovimab	2,933	**BA.2** 1,573 (Sequence-confirmed)	COVID-19 vaccine status ≤ 1: 267 (9.1%) 2: 309 (10.5%) 3: 1,858 (63.4%) ≥ 4: 499 (17.0%)
Young-Xu, et al., 2022 [37] (preprint)	US (all)	Observational comparative effectiveness cohort study COVID-19-related hospitalization or all-cause mortality	Exact matching Multivariable Cox regression analyses	US Department of Veterens Affairs healthcare system	Dec 1, 2021 to May 4, 2022	BA.2 dominant (Mar 16, 2022 to May 4, 2022)	High-risk veterans aged ≥ 18 years, diagnosed with COVID-19	Sotrovimab (S) Untreated (U)	148,214 (14,066 after matching)	**BA.2** Total: 360 (Ecological) March 16 to May 4, 2022	Immunosuppressive disease (matched cohort): S: 999 (35%) U: 3,935 (35%) 3 doses of vaccine (matched cohort): S: 957 (34%) U: 3,820 (34%)
Zaqout et al., 2022 [31] (peer-reviewed)	Qatar (all)	Observational comparative effectiveness cohort study Progression to severe, critical, or fatal COVID-19	Exact matching (1:2) conditional logistic regression Immuno-compromised subgroup analysis	Resident population of Qatar	Oct 20, 2021 to Feb 28, 2022	Omicron BA.2: ~ 60.4% 86.3% Omicron-predominant period (with > 70% BA.2 of Omicron cases)	High-risk patients (based on EUA criteria; with no vaccination considered as an additional eligibility criteria)	Sotrovimab (S) No treatment (N)	S: 519 N: 2845	NR (Ecological)	Two or three vaccine doses S: 366 (70.1%) N: 2187 (76.9%)
Zheng et al., 2022 [38] (preprint at time of search; now peer-reviewed^e^)	UK (England)	Observational comparative effectiveness cohort study Hospitalization due to COVID-19; death from COVID-19	Stratified multiple variable Cox regression Propensity score weighting Cox regression analysis Additional sensitivity analyses to assess robustness of main findings	OpenSAFELY platform	Dec 16, 2021 to Feb 10, 2022 Feb 16, 2022 to May 1, 2022	Omicron BA.2 > 50%^a^	Outpatients with one of the listed high-risk conditions	Sotrovimab (S) Molnupiravir (M)	Total period BA.1 (period 1): 5951 S: 3288 M: 2663 Total period BA.2 (period 2): 7949 S: 5979 M: 1970	**BA.2** S: 5979 M: 1970 (Ecological)	Immunosuppression S: 578 (17.6%) M: 547 (20.5%) Three or more vaccinations S: 2901 (88.2%) M: 2300 (86.4%)
Zheng et al., 2023 [39] (preprint)	UK (England)	Observational comparative effectiveness cohort study COVID-19-related hospitalization or death; all-cause hospitalization or death	Multivariable Cox regression analyses Propensity score weighted Cox regression Additional sensitivity analyses	OpenSAFELY platform	Feb 11, 2022 to Oct 1, 2022	BA.2 dominant (Feb 11 to May 31, 2022) BA.5 dominant (June 1 to October 1, 2022)	High-risk adult outpatients with SARS-CoV-2	Sotrovimab (S) Nirmatrelvir/ritonavir (Nir/Rit) Molnupiravir (M)	Total 7683 S: 2847 Nir/Rit: 4836 M: 802 (exploratory analysis)	NR	Immunosuppression: S: 290 (10.2%) Nir/Rit: 525 (10.9%) ≥ 4 vaccines: S: 1,258 (44.2%) Nir/Rit: 2,047 (42.3%)

*AIFA* Agenzia Italiana del Farmaco [Italian medicines agency], *EUA* Emergency Use Authorization, *HR* hazard ratio, *M* molnupiravir, *mAb* monoclonal antibody, *NHS* National Health Service, *NR* not reported, *nir/rit* nirmatrelvir/ritonavir, *R* remdesivir, *Rx* therapy, *S* sotrovimab, *U* untreated, *UKHSA* UK Health Security Agency

^a^Zheng et al. 2022. According to UK Health Security Agency 2022

^b^Patel et al. 2022. A post-hoc analysis of patients diagnosed or treated between June 1, 2022 and July 31, 2022 was also carried out

^c^Nose et al. 2022. Defined as those needing oxygen or ventilation, needing ICU for exacerbation, transferred for hospitalization for exacerbation, or death due to exacerbation

^d^Nose et al. 2022. Variant information was only available for 21/346 patients; therefore, BA.2 prevalence is likely to be underestimated

^e^The number of included patients (and, therefore, the results) are different in the peer-reviewed paper compared with the pre-print

Up to February 27, 2023, seven of the 14 studies were published in an international peer-reviewed journal [26–32], and seven were published as pre-prints [33–39]. Three of the preprints have since been published in a peer-reviewed journal [40, 41, 42]. Studies reported on populations from the US (*n* = 2), UK (*n* = 6), Italy (*n* = 1), Denmark (*n* = 1), France (*n* = 1), Japan (*n* = 2), and Qatar (*n* = 1).

Seven studies were conducted via secondary analyses of healthcare data, with sources including OpenSAFELY [38, 39], Discover-NOW dataset [36], SAIL Databank [33], and the Hospital Episode Statistics database [35]. Other data sources included patient electronic medical records or charts [27, 28, 32, 37], insurance claims [26], and laboratory data [29].

All studies evaluated clinical outcomes associated with sotrovimab use. Four studies compared the effectiveness of sotrovimab relative to untreated control groups or no mAb treatment [26, 31, 33, 37]. Two provided comparative effectiveness data for sotrovimab relative to other treatments (e.g., mAbs, antivirals, corticosteroids) [38, 39]. Four studies comprised a single-arm treatment design and compared clinical outcomes of sotrovimab-treated patients during BA.2 and/or BA.5 predominant periods versus the BA.1 period [30, 32, 34, 35]. Descriptive reporting rates of clinical outcomes (e.g. hospitalization) in sotrovimab-treated patients were used in five studies [27–29, 32, 36].

As all studies were observational, sotrovimab was utilized as standard of care in accordance with local guidelines. For the studies in the US, UK, Italy, France, Japan and Qatar, sotrovimab 500 mg was the label recommended dose at the time of the study period. We cannot exclude that another dosage was used for the study in Denmark.

Nine studies reported outcomes for sotrovimab during both Omicron BA.1 and BA.2 predominance [26, 29, 30, 32–34, 36–38]. One study reported outcomes during periods of Omicron BA.1, BA.2 and BA.5 predominance [36], two studies during periods of Omicron BA.2 and BA.5 predominance [35, 39], and one Japanese study during periods of Omicron BA.1 and BA.5 predominance [27]. Of note, Cheng et al. also reported clinical outcomes for March and April 2022 when Omicron BA.2 was becoming predominant in the United States, with estimated prevalence of 50% and 100%, respectively [26]. Zaqout et al. only reported outcomes during a period when both Omicron BA.1 and BA.2 were circulating, without differentiating outcomes by subvariant, but during which > 70% of incidence cases were estimated to be BA.2 infections [31].

Eleven of the 14 studies employed an ecological design, with the date or month of COVID-19 diagnosis used as a proxy for the likelihood of an infection being attributable to the prevalent Omicron subvariant circulating in the country/region at the time [26, 27, 30–33, 35–39]. The other three studies used sequencing data to ascertain the SARS-CoV-2 subvariant of infection [28, 29, 34].

Collectively, the 14 studies included over 1.7 million high-risk patients with COVID-19, defined as those with pre-specified comorbid conditions and/or characteristics leading to progression to severe COVID-19 (note that there is a risk of partial study population overlap between observational studies conducted in the same country). Approximately 41,000 patients received sotrovimab as an early treatment for mild-to-moderate COVID-19. Sample size varied between studies, ranging from 179 patients in a single-center study [27] to 1,530,501 patients from a nationwide US insurance claims database [26]. Sample sizes of sotrovimab-treated patients within specific variant predominance periods ranged from *n* = 20–5979 during BA.2 and *n* = 76–1383 during BA.5 predominance. The high-risk populations were heterogeneous, reflecting the differing treatment recommendations in each country at the time of study conduct. As sotrovimab was administered as standard of clinical care, the eligibility criteria for being enrolled in a study reflected the guideline recommendations for sotrovimab as an early COVID-19 treatment in individual countries.

Five studies were conducted in adults aged ≥ 18 years [28, 33, 37–39], eight studies included patients aged ≥ 12 years [26, 29–32, 34–36], and one study did not report the age of patients [27]. The reported mean age of sotrovimab-treated patients in the selected studies ranged from 40 [31] to 79 [27] years.

Of the 14 included studies, seven reported on the composite measure of hospitalization or mortality during Omicron BA.2 and BA.5 predominance, either related to COVID-19 [29, 34, 38, 39] and/or all-cause [26, 33, 34, 39] (Table 3). Three studies reported estimates for mortality alone [27, 30, 38] and four studies reported on hospitalization alone [30, 31, 35, 36]. One study reported on hospitalization or emergency department or urgent care visits [37], and one study briefly reported on the need for intensive care during COVID-19 infection [27]. The Japanese study by Nose et al. included a clinical endpoint of proportion of progressors, defined as patients who required oxygen, non-invasive or invasive ventilation, extracorporeal membrane oxygenation, admission to high care unit or ICU, transfer to another hospital, or died from exacerbation of SARS-CoV-2 infection [32]. In Japan, patients with COVID-19 were routinely hospitalized at the beginning of treatment. This may explain why the studies by Fujimoto et al. [27] and Nose et al. [32] did not report hospitalization rates.

**Table 3:** Clinical effectiveness of sotrovimab during Omicron BA.2 and BA.5 predominance

Variant predominant	Outcome definition	Outcome time point	Sotrovimab (*N*)	Comparator (*N*)	Outcome *N* (%)		Relative effect (95% CI), significance
					Sotrovimab	Comparator	
Cheng et al., 2023 [26]							
Overall (Sept 2021 through Apr 2022)	Hospitalization or mortality (all-cause)	30 days of diagnosis	15,633	No mAb (unmatched: 1,514,868; matched: 62,532)	419 (2.68)	Unmatched: 84,720 (5.59) Matched: NR	RR 0.45 (0.41–0.49), *p* < 0.0001^a^ PS-matched 0.39 (0.35–0.43), *p* < 0.0001^b^
Mar 2022 through Apr 2022	Hospitalization or mortality (all-cause)	30 days of diagnosis	Mar 2022: 1046 Apr 2022: 68 Combined for BA.2: 1,114	No mAb (unmatched Mar 2022: 65,521; Apr 2022: 117,238; combined for BA.2: 182,759; matched: NR)	Mar 2022: 21 (calculated, 2.01% of 1046) Apr 2022: 1 (calculated, 1.47% of 68) Combined for BA.2: 22 (2.0)	Mar 2022: 2,863 (calculated, 4.37% of 65,521) Apr 2022: 2,228 (calculated, 1.90% of 117,238) Combined for BA.2: 5,091 (2.8) Matched: NR	Mar 2022 RR 0.41 (0.27–0.62), *p* < 0.0001^a^ March 2022 PS-matched 0.36 (0.23–0.56), *p* < 0.0001^b^ Apr 2022 RR 0.54 (0.08–3.54), *p* = 0.52^a^ Apr 2022 PS-Matched 0.32 (0.04–2.38), *p* = 0.52^b^
Evans et al., 2023 [33]							
BA.1 and BA.2	All-cause hospitalization or death	28 days of treatment	1,079	Molnupiravir (M) (359) Nirmatrelvir/Ritonavir (Nir/Rit) (602) Untreated (U) (4973)	53 (4.9)	M: 14 (3.9) Nir/Rit: 17 (2.8) U: 544 (10.9)	S: Adjusted HR 0.73 (0.55–0.98) M: Adjusted HR 0.49 (0.29–0.83) Nir/Rit: Adjusted HR 0.59 (0.36–0.97) U: Reference group
Fujimoto et al., 2022 [27]							
BA.5	Mortality	During BA.5 wave	47	Remdesivir/dexamethasone (Rem/Dex) (12)	1 (2.1)	Rem/Dex: 1 (8.3)	NR
BA.5	Required oxygen therapy on first and third day of treatment	First and third day of treatment	47	NR	2 (4.3)	NR	NR
Harman et al., 2023 [34]							
BA.2 vs BA.1	Hospitalization or mortality (all-cause)	14 days of treatment	BA.2 (4565) BA.1 (4285)	–	BA.2: 77 (1.7) BA.1: 91 (2.1)	–	BA.2 vs BA.1 HR 1.17 (0.74–1.86), *p* = NR^c^
BA.2 vs BA.1	Hospitalization or mortality (COVID-19-related)	14 days of treatment	BA.2 (4565) BA.1 (4285)		BA.2: 62 (1.4) BA.1: 73 (1.7)		BA.2 vs BA.1 HR 0.98 (0.58–1.65), *p* = NR^c^
Martin-Blondel et al., 2023 [28]							
BA.1 and BA.2	COVID-19-related hospitalization	28 days of treatment	193	Nirmatrelvir (Nir) (55)	4 (2)	Nir: 0 (0)	NR
BA.1 and BA.2	COVID-19-related deaths	28 days of treatment	193	Nir (55)	0 (0)	Nir: 1 (2)	NR
Mazzotta et al., 2023 [29]							
BA.1	Hospitalization (COVID-19-related) or mortality (all-cause)	30 days of treatment	146	Nirmatrelvir/ ritonavir (Nir/Rit) (49) Remdesivir (R) (84) Molnupiravir (M) (99)	5 Overall BA.1 + BA.2: 7/226 (3.1)	Nir/Rit: 2 Overall BA.1 + BA.2: 2/87 (2.3) R 0 (0) M 0 (0)	NR
BA.2	Hospitalization (COVID-19-related) or mortality (all-cause)	30 days of treatment	56	Nir/Rit (35) R (34) M (18)	2 Overall BA.1 + BA.2: 7/226 (3.1)	Nir/Rit: 0 Overall BA.1 + BA.2: 0/87 R 0 (0) M 0 (0)	NR
Nose et al., 2022 [32]							
BA.1	Progression	29 days of treatment	118	NR	1 (0.8)	NR	(0.02–4.63)
BA.2	Progression	29 days of treatment	128	NR	0 (0.0)	NR	(0.00–2.84)
Patel et al., 2022 [36]							
BA.1, BA.2 and BA.5	COVID-19-related hospitalization	28 days of treatment	696	Nirmatrelvir/ritonavir (Nim/Rit) (337) Molnupiravir (M) (470) Untreated (U) (4044)	5 (0.7)	Nim/Rit: < 5 (0.3–1.2) M: 10 (2.1) U: 114 (2.8)	NR
BA.1, BA.2 and BA.5	All-cause hospitalization	28 days of treatment	696	Nirmatrelvir/ritonavir (Nim/Rit) (337) Molnupiravir (M) (470) Untreated (U) (4,044)	35 (5.0)	Nim/Rit: 5 (1.5) M: 19 (4.0) U: 251 (6.2)	NR
BA.1, BA.2, BA.5	Mortality	28 days of treatment	696	Nim/Rit (337) M (470) U (4,044)	8 (1.1)	Nim/Rit: < 5 (0.3–1.2) M: 7 (1.5) U: 75 (1.9)	NR
BA.2	COVID-19-related hospitalization	During period of pre-dominance	415	Nim/Rit (269) M (59) U (1,302)	< 5 (0.2–1.0)	Nim/Rit: NR M: < 5 (1.7–6.8) U: 27 (2.1)	NR
BA.5	COVID-19-related hospitalization	During period of pre-dominance	197	Nim/Rit (228) M (13) U (657)	< 5 (0.5–2.0)	Nim/Rit: 0 (0) M: < 5 (7.7–30.8) U: 12 (1.8)	NR
Patel et al., 2023 [35]							
BA.1, BA.2 and BA.5	COVID-19-related hospitalization	28 days of treatment	10,096	NR	96 (1.0)	NR	NR
BA.1, BA.2 and BA.5	All-cause hospitalization	28 days of treatment	10,096	NR	465 (4.6)	NR	NR
BA.1, BA.2 and BA.5	Mortality	28 days of treatment	10,096	NR	27 (0.3)	NR	NR
BA.2	COVID-19-related hospitalization	28 days of treatment	3884	NR	37 (1.0)	NR	IRR 0.76 (0.44–1.30), *p* = 0.31
BA.5	COVID-19-related hospitalization	28 days of treatment	1383	NR	10 (0.7)	NR	IRR 0.56 (0.26–1.19), *p* = 0.13
Rasmussen et al., 2023 [30]							
Delta, BA.1 and BA.2	Hospitalization	90 days of treatment	2933	NR	813 (27.7)	NR	NR
Delta, BA.1 and BA.2	Mortality	90 days of treatment	2933	NR	156 (5.3)	NR	NR
2022 (surrogate for Omicron) vs 2021 (surrogate for Delta)	Hospitalization	90 days of treatment	NR	NR	NR	NR	Adjusted HR 0.86 (0.71–1.04)^g^
2022 (surrogate for Omicron) vs 2021 (surrogate for Delta)	Mortality	90 days of treatment	NR	NR	NR	NR	Adjusted HR 0.64 (0.44–0.95)^g^
Young-Xu et al., 2022 [37]							
BA.2	COVID-19-related hospitalization, emergency department or urgent care visits	30 days of treatment	74	Untreated (U) (286)	< 10 (4.0)	31 (10.8)	Adjusted HR 0.29 (0.08–0.98)^g^
Zaqout et al., 2022 [31]							
Delta and Omicron	Progression to severe, critical, or fatal COVID-19	NR	345	No treatment (583)	4 (1.2)	3 (0.5)	Adjusted OR 2.67 (0.60–11.91)^d^
Delta and Omicron	Progression to severe, critical, or fatal COVID-19 in patients at higher risk of severe COVID-19^e^	NR	295	No treatment (533)	3 (1.0)	8 (1.5)	Adjusted OR 0.65 (0.17–2.48)^d^
Omicron	Progression to severe, critical, or fatal COVID-19	NR	233	No treatment (431)	2 (0.9)	0 (0)	NR
Omicron	Progression to severe, critical, or fatal COVID-19 in patients at higher risk of severe COVID-19^e^	NR	210	No treatment (391)	2 (1.0)	4 (1.0)	0.88 (0.16–4.89)^d^
Zheng et al., 2022^i^ [38]							
BA.1	Hospitalization or mortality (COVID-19-related)	28 days of treatment	3331	Molnupiravir (2689)	32 (0.96)	55 (2.05)	Stratified Cox HR 0.54 (0.33–0.88), *p* = 0.014^f^ PSW-Cox HR 0.50 (0.31–0.81), *p* = 0.005^f^
BA.2	Hospitalization or mortality (COVID-19-related)	28 days of treatment	5979	Molnupiravir (1970)	57 (0.95)	40 (2.03)	Stratified Cox HR 0.44 (0.27–0.71), *p* = 0.001^f^ PSW-Cox HR 0.53 (0.32–0.86), *p* = 0.010^f^
BA.1	Mortality (COVID-19-related)	28 days of treatment	3331	Molnupiravir (2689)	7 (0.21)	18 (0.67)	NR
BA.2	Mortality (COVID-19-related)	28 days of treatment	5979	Molnupiravir (1970)	9 (0.15)	19 (0.96)	NR
Zheng et al., 2023 [39]							
BA.2 and BA.5	COVID-19-related hospitalization or death	28 days of treatment	2847	Nirmatrelvir/Ritonavir (Nir/Rit) (4,836)	19 (0.67)	Nir/Rit: 33 (0.68)	Stratified Cox HR 1.14 (0.62–2.08), *p* = 0.673^ h^ PSW-Cox HR 0.88 (0.45–1.71), *p* = 0.700^ h^
BA.2 and BA.5	All-cause hospitalization or death	28 days of treatment	2847	Nir/Rit (4,836)	97 (3.41)	123 (2.55)	Stratified Cox HR 0.89 (0.67–1.18), *p* = 0.412^ h^ PSW-Cox HR 0.84 (0.63–1.13), *p* = 0.248^ h^
BA.2 and BA.5	Mortality	28 days of treatment	2847	Nir/Rit (4,836)	≤ 5 (≤ 0.18)	8 (0.17)	NR

*aOR* adjusted odds ratio, *BMI* body mass index, *HIV* human immunodeficiency disease, *HR* hazard ratio, *IMD* indices of multiple deprivation, *IRR* incidence rate ratio, *NR* not reported, *PS* propensity score, *PSW* propensity score weighted, *RR* relative risk

^a^Adjusted for diagnosis month category, age, gender, region, rurality, high-risk conditions, documented COVID-19 vaccine

^b^Matched on diagnosis month, age, gender, region, rurality, and selected high-risk conditions

^c^Hospitalization excluded hospital admissions for injury-related reasons. Adjusted for age group, linear effect in age and vaccination status, to account for confounders

^d^Cases and controls were exact-matched one-to-two by vaccination status, prior infection status, sex, age group, nationality group, comorbidity count, and epidemic phase

^e^Defined as individuals who were immunocompromised (recipients of solid organ or hematopoietic stem cell transplant, patients receiving chemotherapy or immunosuppressive treatments, patients with severe immunodeficiency, and patients with HIV), unvaccinated individuals, those aged ≥ 75 years, and pregnant women

^f^Adjusted for age, sex, ten high risk cohort categories, ethnicity, IMD quintiles, vaccination status, calendar week, BMI category, diabetes, hypertension, chronic cardiac and respiratory diseases

^g^Variables adjusted for not reported in publication

^h^Adjusted for age, sex, high risk cohort categories, ethnicity, IMD quintiles, vaccination status, calendar date, BMI category, diabetes, hypertension, chronic cardiac and respiratory disease

^i^The number of included patients (and, therefore, the results) are different in the peer-reviewed paper compared with the pre-print

Clinical outcomes were generally reported within 28–30 days of treatment, with the exception of Harman et al. (which reported outcomes within 14 days of treatment [34]) and Rasmussen et al. (which reported outcomes within 90 days of COVID-19 diagnosis [30]).

One study (from Qatar) described the results for progression to severe, critical, or fatal COVID-19 [31]. It should be noted that the reasons for COVID-19-related hospital admission in Qatar differed from other included studies. Hospitalization was unrelated to COVID-19 severity and was utilized as a means for dispensing treatment, or as part of a proactive approach to prevent transmission and spread of the disease, as opposed to reducing the risk of further progression [43]. As such, any comparison of hospitalization rates with the other studies should be considered with caution.

### Quality assessment

**Fig. 2:**
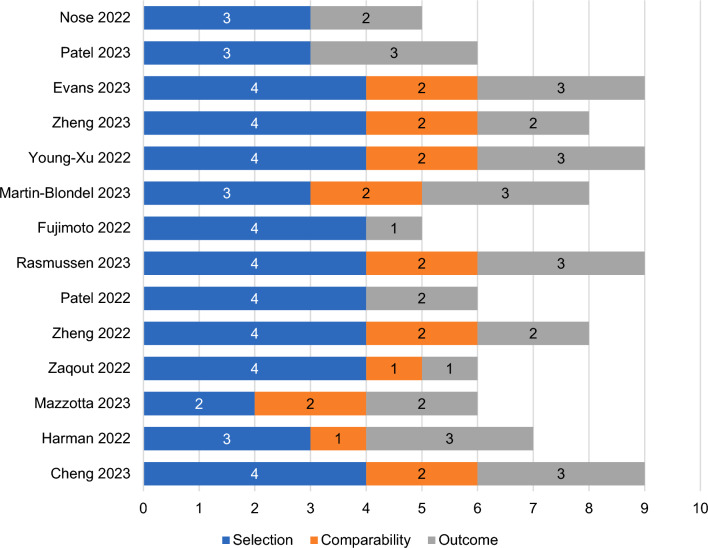
NOS total and bias domain scores across the studies included in the SLR. *NOS* Newcastle Ottawa scale, *SLR* systematic literature review

Out of the total maximum attainable score of nine on the NOS, eight studies achieved a score of ≥ 7 [26, 28, 30, 33, 34, 37–39], suggesting that they were of comparatively good quality (Fig. 2). The remaining studies were awarded a score of 6 [29, 31, 35, 36] or 5 [27, 32]. Mazzotta et al. was primarily designed to explore changes in SARS-CoV-2 viral load following treatment [29], and its score of six mainly reflects shortcomings in assessing clinical outcomes rather than overall study quality.

All studies scored 3 or 4 on the selection bias domain (out of a maximum score of 4), except Mazzotta et al. (score of 2), for which the ascertainment of exposure to sotrovimab was not clearly stated [29]. Most of the studies (*n* = 8/14) scored 2 on the comparability bias domain (out of a maximum score of 2), reporting no major differences in the baseline characteristics of patients or providing adjustment analyses. An exception was Nose et al., which scored zero on this domain due to being a single-arm study.

NOS was not used to assess more specifically the quality of information related to the effectiveness of sotrovimab during Omicron BA.2 or BA.5 predominance. This is of particular relevance to Cheng et al. [26] and Zaqout et al. [31], which report limited data on Omicron BA.2.

#### Summary of clinical outcomes

The clinical outcomes data extracted from the 14 studies included in this review are provided in Table 3.

**Fig. 3:**
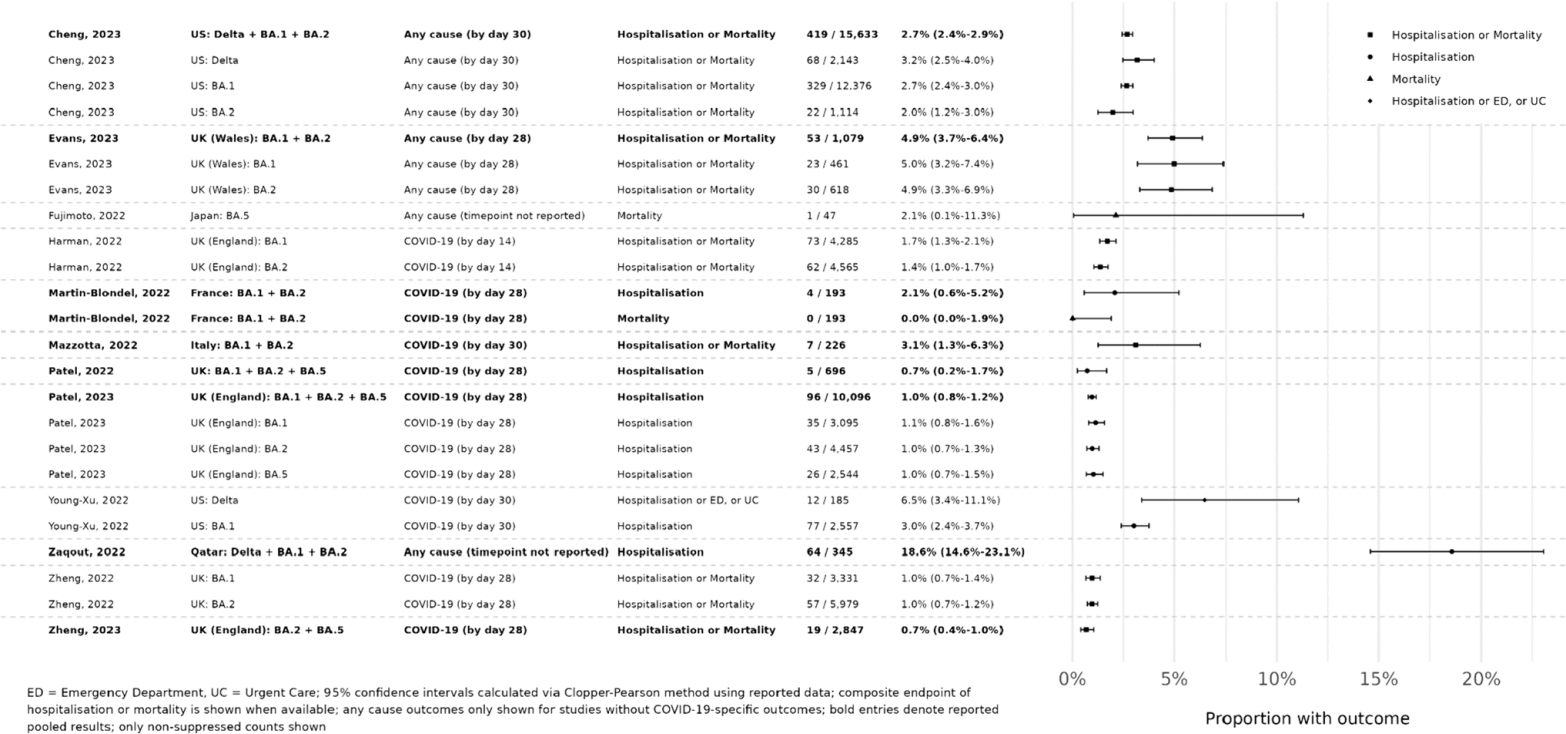
Point estimates for hospitalization or mortality (as a composite endpoint) or clinical progression for sotrovimab-treated patients. Rasmussen et al. [30] not included as hospitalization and mortality outcomes are reported at day 90, rather than 28- or 30-day period used to define acute COVID-19 outcomes in other studies. Nose et al. [32] not included as the study outcome and source population (the proportion of people who were hospitalized who are still hospitalized at day 29) are not aligned with other studies.

Rates of COVID-19-related hospitalization or mortality were consistently low across all studies and during periods of Omicron BA.2 and BA.5 predominance (Table 3; Fig. 3). For sotrovimab-treated patients, rates of COVID-19-related hospitalization or death ranged from 0.95% [38] to 4.0% [37] during Omicron BA.2 predominance and from 0.5 to 2.0% during BA.5 predominance [36].

The proportions of patients experiencing all-cause hospitalization and/or mortality ranged between 1.7 and 2.0% for the Omicron BA.2 period, as reported by Harman et al. (day 14) and Cheng et al. (day 30), respectively [26, 33, 34]. Only one study (Zheng et al.) reported a composite of all-cause hospitalization and/or death in sotrovimab-treated patients during the BA.5 predominance period [39]; the reported rate (3.4%) was combined with the BA.2 period [39].

Zheng et al. reported a COVID-19-related mortality rate of 0.15% during Omicron BA.2 predominance for patients treated with sotrovimab (*n* = 9/5979), versus 0.96% for patients treated with the antiviral molnupiravir (*n* = 19/1970) [38]. COVID-19-related mortality during the combined BA.2 and BA.5 predominance periods was estimated at ≤ 0.18% for the sotrovimab group (*n* =  ≤ 5/2847) vs 0.17% for nirmatrelvir/ritonavir (*n* = 8/4836) [39], while all-cause mortality during BA.5 predominance was estimated at 2.1% (*n* = 1/47) for the sotrovimab group vs 8.3% (*n* = 1/12) for remdesivir + dexamethasone [27].

#### Clinical effectiveness of sotrovimab vs control (untreated or no mAb)

Four studies examined the clinical effectiveness of sotrovimab vs a control during Omicron BA.2 predominance [26, 31, 33, 37].

The US-based study by Cheng et al. reported that sotrovimab was associated with a lower risk of 30-day all-cause hospitalization or mortality compared with no mAb treatment during March and April 2022 (BA.2 period; Table 3) [26]. In March 2022, sotrovimab treatment (*n* = 1046) resulted in a significant reduction in propensity score-matched relative risk (RR) of 64% (adjusted RR 0.36, 95% CI 0.23–0.56; *p* < 0.001) in 30-day all-cause hospitalization or mortality vs patients not treated with a mAb. In April 2022, the propensity score-matched RR reduction was 68% (adjusted RR 0.32, 95% CI 0.04–2.38; *p* = 0.519) compared with patients not treated with a mAb.

The Zaqout et al. study in Qatar reported that the overall (periods of Delta and Omicron predominance combined) adjusted odds ratio (OR) of disease progression to severe, critical or fatal COVID-19 for the exact-matched sotrovimab-treated versus untreated control group was 2.67 (95% CI 0.60–11.91; Table 3) [31]. An adjusted OR of disease progression during the Omicron-dominated time period could not be calculated as none of the 431 untreated patients were observed to have progressed; two of the 233 (0.9%) sotrovimab treated-patients progressed during this phase. In the same study, among patients described as being at higher risk of severe forms of COVID-19 (immunocompromised, unvaccinated individuals, aged ≥ 75 years, and pregnant women) sotrovimab-treated patients had lower odds of progression compared with untreated patients (adjusted OR 0.65, 95% CI 0.17–2.48). Restricting the analysis to the Omicron-predominant period (December 19, 2021 to February 28, 2022) for the subgroup of higher-risk patients yielded an adjusted OR of 0.88 (95% CI 0.16–4.89) (Table 3).

In the US study by Young-Xu et al., treatment with sotrovimab during BA.2 predominance was associated with a reduced risk of COVID-19-related hospitalization, emergency department, or urgent care visits (*n* =  < 10/74) within 30 days vs the exact-matched untreated control group (*n* = 31/286; adjusted hazard ratio [HR] 0.29 [95% CI 0.08–0.98]; Table 3) [37]. During the BA.1 period, the adjusted HR of 30-day COVID-19-related hospitalization or all-cause mortality in the sotrovimab group (*n* = 92/2557) vs the group that received no treatment (*n* = 735/10,297) was 0.30 (95% CI 0.23–0.40).

In a UK study by Evans et al., the adjusted HR of all-cause hospitalization or death within 28 days during the study period (BA.1 and BA.2 predominant periods combined) was reported as 0.73 (95% CI 0.55–0.98) for unmatched sotrovimab vs untreated control groups (Table 3) [33].

#### Clinical effectiveness of sotrovimab vs active comparators

Compared with molnupiravir, sotrovimab was associated with a lower risk of COVID-19-related hospitalization or death during the BA.2 predominance period in England (February 16 to May 1, 2022), after adjusting for demographics, high-risk cohort categories, vaccination status, calendar time, BMI, and other comorbidities (adjusted HR 0.44, 95% CI 0.27–0.71; *p* = 0.001; propensity score weighted Cox model, adjusted HR 0.53, 95% CI 0.32–0.86, *p* = 0.01) [38].

During the BA.2 (February 11 to May 31, 2022) and BA.5 (June 1 to October 1, 2022) predominance periods in England, treatment with nirmatrelvir/ritonavir was associated with a similar risk of COVID-19-related hospitalization or death to sotrovimab (adjusted HR 1.35, 95% CI 0.54–3.34, and 0.74, 95% CI 0.31–1.78, respectively, using a fully-adjusted stratified Cox model) [39].

#### Comparison of clinical outcomes between periods of different circulating variants

Five studies compared clinical outcomes following sotrovimab treatment during the Omicron BA.1 period and the BA.2 and/or BA.5 predominance periods (Table 3) [30, 32–35].^5^

In the Harman et al. study in England, risk of hospital admission with a length of stay of ≥ 2 days within 14 days of community treatment with sotrovimab showed no statistically significant difference between BA.1 (2.1%, *n* = 91/4285) and BA.2 (1.7%, *n* = 77/4565) (HR 1.17, 95% CI 0.74–1.86) [34]. Rasmussen et al. reported no difference in risk of all-cause mortality and all-cause hospitalization (≥ 24 h within 90 days of COVID-19 diagnosis) between Omicron BA.2 (*n* = 1573) and BA.1 (*n* = 381) subvariants in patients in Denmark treated with sotrovimab (adjusted HR 1.04, 95% CI 0.84–1.29 for all-cause hospitalization; adjusted HR 1.04, 95% CI 0.59–1.83 for mortality) [30]. Similarly, in a subanalysis of the study by Evans et al., all-cause hospitalization or death rates among patients in the UK treated with sotrovimab during the BA.1 and BA.2 periods were similar (5.0% vs 4.9%, respectively), with no significant difference between the subvariant time periods (HR 0.76 [95% CI 0.50–1.18] vs. 0.70 [95% CI 0.48–1.03], respectively) [33]. In another UK study, Patel et al. reported no difference in risk of COVID-19-related hospitalization during the Omicron BA.2 (1.0%) and BA.5 (0.7%) predominance periods vs the BA.1 (1.0%) phase among patients treated with sotrovimab [incidence rate ratio (IRR) 0.76, 95% CI 0.44–1.30, *p* = 0.31, and 0.56, 95% CI 0.26–1.19, *p* = 0.13, respectively) [35]. In an interim analysis of a Japanese study, Nose et al. reported similar rates of progression for sotrovimab-treated patients infected with Omicron BA.1 (0.8%; *n* = 1/118, 95% CI 0.02–4.63) and BA.2 (0%; 0/128, 95% CI 0.00–2.84), suggesting consistent clinical benefit with sotrovimab during the BA.2 predominant period [32].

## Discussion

This SLR identified and assessed all observational studies in the available literature from January 1, 2022 to February 27, 2023 that reported clinical outcomes for patients treated with sotrovimab during Omicron BA.2 and BA.5 predominance. These studies consistently reported low rates of all-cause or COVID-19-related hospitalization or death in high-risk, non-hospitalized patients receiving early treatment with sotrovimab 500 mg.

These findings confirm and extend those of our recently published SLR, which reviewed clinical outcomes of patients with COVID-19 treated with sotrovimab 500 mg during BA.2 subvariant predominance, and reported consistently low proportions of severe clinical outcomes (such as hospitalization and mortality) in sotrovimab-treated patients during BA.1 and BA.2 predominance [21]. Our first review was conducted shortly after the emergence of the BA.2 subvariant (which was the first to show reduced susceptibility to sotrovimab in vitro [18]), and therefore only a limited number of real-world studies of sotrovimab effectiveness againnt BA.2 were available. The current SLR includes additional studies from the BA.2 predominant period that add substantially to the evidence base, including a robust study conducted using the OpenSAFELY platform [39]. It also includes studies from the BA.5 predominant period; since studies have also suggested reduced susceptibility of this subvariant to sotrovimab in vitro [18], it was important to assess the real-world effectiveness of sotrovimab during the BA.5 predominant period so that clinicians have the best available evidence (in the absence of data from clinical trials). Another recent SLR and meta-analysis demonstrated the real-world effectiveness of sotrovimab for reducing hospitalization and mortality during both the Delta and Omicron BA.1 periods of predominance [20].

Of the 14 studies included in this SLR, six high-quality studies addressed the clinical effectiveness of sotrovimab during periods of BA.2 or BA.5 predominance [30, 33, 34, 37–39]. Of these, two multicenter studies from the US [37] and UK [33] reported a lower risk of COVID-19-related hospitalization, emergency department or urgent care visits, and all-cause hospitalization or death with sotrovimab vs no treatment during BA.2 predominance in both countries. These findings support the maintained clinical effectiveness of sotrovimab against the BA.2 subvariant. In addition, although only a limited number of studies identified in our review were conducted during the period of BA.5 predominance, the findings from these four studies demonstrated low rates of COVID-19-related and all-cause clinical outcomes in sotrovimab-treated patients during this time [27, 35, 36, 39]. Three studies (one from Denmark and two from England) statistically compared clinical outcomes of sotrovimab-treated patients between the BA.1 and BA.2 or BA.5 predominance periods [30, 34, 35]. Each found no difference in the risk of all-cause or COVID-19-related hospitalization or death during BA.2 and BA.5 predominance compared with BA.1.

Only two of the studies included in this review were conducted in the US [26, 37]. Both studies evaluated sotrovimab effectiveness during the BA.1 and BA.2 predominant periods. No data after the emergence of BA.2 were generated in the US since sotrovimab use was discontinued after April 2022 when prevalence of the BA.2 subvariant was above 50%. Consequently, all data from the BA.5 period are derived from outside the US, mainly in Europe.

Two observational cohort studies by Zheng et al. leveraged the substantial size of the OpenSAFELY platform database across BA.2 and BA.5 subvariant periods using propensity scoring methodology with sensitivity analyses to support the robustness of the data [38, 39]. In the earlier of these two studies, sotrovimab 500 mg was associated with a substantially lower risk of 28-day COVID-19-related hospitalization or death during the Omicron BA.2 subvariant surge compared with molnupiravir after adjusting for demographics, high-risk cohort categories, vaccination status, calendar time, BMI and other comorbidities (*n* = 1970) [38]. Rates of COVID-19-related hospitalization or death for sotrovimab were comparable across the Omicron BA.1 (0.96%) and BA.2 (0.95%) periods, and mortality was lower in patients treated with sotrovimab vs molnupiravir during both periods [38]. It should be noted, however, that between the Omicron BA.1 and BA.2 periods, guidance in the UK for molnupiravir was changed from a second- to third-line treatment option, while sotrovimab remained a first-line option during both periods [44]. Although the effect of this change is unclear, it may have impacted the baseline characteristics of patients who received molnupiravir; the authors acknowledge the risk of bias is small [38].

More recently, the authors reported no difference in the risk of COVID-19-related hospitalization or death between nirmatrelvir/ritonavir- and sotrovimab-treated patients during BA.2 and BA.5 predominance [39]. The authors concluded that these data support a protective role of sotrovimab treatment against the Omicron BA.2 and BA.5 subvariants [38, 39].

The results from Zheng et al. are further supported by the large retrospective cohort studies conducted by Harman et al. [34] and Patel et al. [36]. In Harman et al., variant sequencing data from patients in England were used to assess the risk of hospital admission within 14 days in patients treated with sotrovimab and infected with Omicron BA.2, compared with Omicron BA.1. Similar to Zheng et al. [38], no significant difference in clinical outcomes was observed between BA.2 and BA.1 subvariants. The consistent results of Harman et al. and Zheng et al., despite assessment of different clinical outcomes and across overlapping populations, further support the robustness of these findings. In Patel et al., consistently low COVID-19-related hospitalization rates were observed among patients receiving sotrovimab, with no evidence of significant differences in incidence rate ratio for any period compared with BA.1 [36].

## Limitations

There are some limitations to this study, which should be discussed. Firstly, the number of studies identified in this SLR is small, although they collectively included over 1.7 million high-risk participants. The COVID-19 landscape is also rapidly evolving and real-world data for sotrovimab during BA.2 and BA.5 predominance and onwards is still emerging. Further evidence has been published since we completed our literature search, including an OpenSAFELY population-based cohort analysis demonstrating a reduced risk of adverse outcomes among sotrovimab-treated patients versus no treatment in England during the BA.1 and BA.2 periods [45]. In addition, a comparative effectiveness study using the DISCOVER dataset (north-west London) assessed the risk of 28-day COVID-19-related hospitalisation and/or COVID-19-related death among highest-risk patients who received sotrovimab or no early COVID-19 treatment [46]. The risk of hospitalisation and/or death was lower for the sotrovimab-treated cohort across periods of BA.1, BA.2, and BA.5 predominance, although statistical significance was reached only for the BA.1 period. Additional observational studies will further contribute to the understanding of sotrovimab’s effectiveness during recent Omicron subvariant periods. Furthermore, seven studies published in preprint databases have been included in this SLR [33–39]. While these should be interpreted with caution as they are not peer-reviewed, preprint publication has been commonly used throughout the COVID-19 pandemic as a means of rapidly reporting outcomes to guide responsive public health decision-making [47].

The observational nature of the studies included has inherent limitations, such as lack of a randomized design; however, this limitation was mitigated in many studies by use of appropriate measures to control for confounding factors. Also, there are a number of factors that can influence outcomes following infection with SARS-CoV-2 viral variants, including immunocompromised status, previous vaccination, and previous viral infection. In conducting a SLR, we were reliant on the type and quality of information reported in the individual studies; most of the included studies reported (to some degree) the immunocompromised status and extent of previous vaccination among participants, however, there was no consistency across studies in how these data were reported, and we were unable to unpick the potential impact of these factors as part of this SLR.

Viral sequencing to confirm the infecting variant or subvariant is rarely done as standard of care and, therefore, rarely reported in real-world studies. Due to a lack of sequencing data, most of the studies included in this SLR used an ecological design to infer the infecting variant using the date of SARS-CoV-2 infection [26, 27, 30, 31, 33, 35–39]. Exceptions were Mazzotta et al. and Harman et al., which used sequencing data to fully ascertain the SARS-CoV-2 subvariant of infection [29, 34]. The use of an ecological design by most of the included studies means definitive conclusions cannot be drawn on the effectiveness of sotrovimab against the BA.2 and BA.5 subvariants.

Finally, a meta-analysis was not considered feasible as the included studies were diverse in terms of population, endpoints, study design, and analytical methods used to estimate clinical outcomes during Omicron BA.2 or BA.5. Combining studies is unwise as this may amplify the presence of confounding factors.

## Conclusions

Results from this SLR build on the findings from our earlier published review, providing further evidence for continued clinical effectiveness of early treatment with sotrovimab 500 mg IV in preventing severe clinical outcomes during Omicron BA.2 and BA.5 periods vs control/comparators and vs the Omicron BA.1 period among high-risk, non-hospitalized patients. The studies included in this review were consistent in reporting similarly low proportions of severe clinical outcomes (such as hospitalization and mortality) in sotrovimab-treated patients between the periods of Omicron BA.1, BA.2 and BA.5 subvariant predominance. Additional observational studies are warranted to contribute to the understanding of real-world effectiveness of sotrovimab against Omicron BA.2 and BA.5 subvariants, as well as future evolving variants.

## Supplementary Information

Below is the link to the electronic supplementary material.

Supplementary file1 (DOCX 109 KB)

## Data Availability

All datasets generated for this study are included in this manuscript.
